# Anticardiolipin and anti‐beta 2‐glycoprotein I antibodies in patients with unexplained articular manifestations

**DOI:** 10.1002/jcla.24812

**Published:** 2022-12-13

**Authors:** Sarra Melayah, Mariem Ghozzi, Ibtissem Ghedira, Amani Mankaï

**Affiliations:** ^1^ Immunology Laboratory Farhat Hached Hospital Sousse Tunisia; ^2^ Faculty of Pharmacy Monastir University Monastir Tunisia; ^3^ Resarch Unit LR12SP11 on "Biologie moléculaire appliquée aux maladies cardiovasculaires et neurologiques, aux néphropathies héréditaires et à la pharmacogénétique" Biochemistry Department Sahloul University Hospital Sousse Tunisia; ^4^ Research Laboratory for "Epidemiology and Immunogenetics of Viral Infections, LR14SP02" Sahloul University Hospital Sousse Tunisia; ^5^ Higher School of Health and Technical Sciences Tunis El Manar University Tunis Tunisia; ^6^ Research Unit UR18ES01 on "Obesity: etiopathology and treatment" National Institute of Nutrition and Food Technology Tunis Tunisia

**Keywords:** anti‐beta 2‐glycoprotein I antibodies, antiphospholipid antibodies, arthralgia, arthritis

## Abstract

**Objective:**

To determine the frequency of antiphospholipid antibodies (aPL) in patients with unexplained articular manifestations.

**Material and Methods:**

Three hundred thirteen patients suffering from arthritis or arthralgia without evident cause and 266 healthy blood donors (HBD) were included in the study. Anticardiolipin antibodies (aCL) and anti‐beta 2‐glycoprotein I antibodies (aβ2GPI) were measured by ELISA.

**Result:**

Out of the 313 patients, 250 were females and 63 were males. The mean age of patients was 49 ± 14 years (17–87 years). One hundred eleven patients have arthralgia and 202 have arthritis. The frequency of aCL and/or aβ_2_GPI (24.9%) was significantly higher in patients than in HBD (10.9%). The frequency of aβ2GPI was 23.6% in patients and 9.4% in the control group (*p* < 10^−3^). aβ2GPI‐IgA was significantly more frequent in patients than in the control group (20.4% vs. 7.5%, *p* < 10^−3^). aβ2GPI was most commonly observed than aCL in patients (23.6% vs. 6.4%, *p* < 10^−6^). IgA isotype of aβ2GPI was the most frequent in 20.4% of patients while IgG and IgM were detected in 5.4% and 2.9% respectively.

**Conclusion:**

This study showed that aPL were common in patients with articular manifestations and were mainly directed against β_2_GPI. The role of these antibodies remains to be specified.

## Graphical abstract

Three hundred thirteen patients suffering from arthritis or arthralgia without evident cause and 266 healthy blood donors (HBD) were included in the study. Anticardiolipin antibodies (aCL) and anti‐beta 2‐glycoprotein I antibodies (aβ2GPI) were measured by ELISA. The frequency of aβ2GPI was significantly higher in patients than in the control group (23.6% vs. 9.4%, *p* < 10^−3^). IgA was the most frequent isotype of aβ2GPI (20.4%) in the patient population.

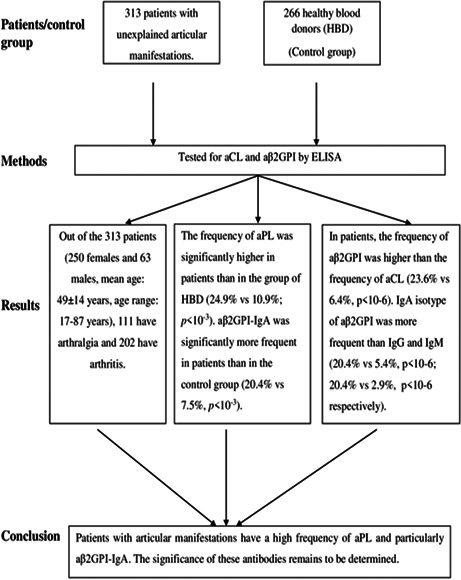

## 1 INTRODUCTION

Antiphospholipid antibodies (aPL) represent a complex and heterogeneous group of antibodies directed against anionic phospholipids or protein‐phospholipid complexes.^1^ Persistent aPL have been associated with antiphospholipid syndrome (APS), which is defined by the presence of recurrent venous and/or arterial thrombosis and often pregnancy morbidity.^2^ Three aPL are listed in the 2006 APS classification criteria,^3^ IgG and IgM anticardiolipin (aCL) and anti‐ beta 2‐glycoprotein I (aβ2GPI) and lupus anticoagulant (LA). “Non‐criteria” antibodies exist as well, in particular, aβ2GPI‐IgA which have aroused greater interest in the last few years. In fact, this isotype of aβ2GPI has been included in the classification criteria for systemic lupus erythemathosus (SLE) since 2012. The value of IgA aβ2GPI in APS diagnosis is still controversial. Some studies support their clinical relevance in APS^4^, ^5^, ^6^ while others failed to demonstrate an added value of this isotype.^7^, ^8^ Otherwise, aβ2GPI‐IgA have been linked to atherosclerotic disease,^9^ chronic renal disease,^10^ early mortality after heart transplantation^11^ and cardiovascular mortality in hemodialysis patients.^12^ aβ_2_GPI‐IgA have also been observed in many autoimmune diseases (AID) other than APS including rheumatoid arthritis (RA),^13^ autoimmune hepatitis,^14^ celiac disease,^15^ primary biliary cholangitis,^16^ and in infectious diseases like hepatitis C^17^ and coronavirus disease 2019 (COVID‐19).^18^ All these autoimmune and infectious diseases may include rheumatic manifestations.

Joint pain is the most common chronic pain making it one of the largest causes of disabilities in the world.^19^ Arthritis is a frequent condition that causes oedema, redness, heat, loss of function and pain. It can affect one or more joints and there exist more than 100 different types.^19^ Since we have previously reported the frequency of aβ2GPI and aCL in RA^13^ and that the frequency of aPL in patients with unexplained arthralgia or arthritis has not been reported previously, we aimed, in this study, to evaluate the frequency of aβ2GPI and aCL in patients suffering from articular manifestations (arthritis or arthralgia) without evident cause.

## 2 MATERIALS AND METHODS

### 2.1 Study population

We conducted a retrospective study including 313 patients suffering from arthritis or arthralgia without evident cause. Serum samples were collected from January 2017 to December 2019 from the database of our Immunology laboratory. We excluded all patients suffering from specific arthritis, rheumatoid arthritis, SLE, psoriatic arthritis or ankylosing spondylitis. Antinuclear antibodies (ANA), rheumatoid factors (RF) and anti‐cyclic citrullinated peptides antibodies (CCP‐Ab) were negative for all patients. Sera of 266 healthy blood donors (HBD) were included as normal controls.

Approval of the study was obtained from the ethical committee of hospital Farhat Hached.

### 2.2 Anticardiolipin antibodies assays

aCL‐IgG and IgM were detected using an enzyme‐linked immunosorbent assay (ELISA) kit (#515, Orgentec Diagnostika®). aCL‐IgA were assessed by ELISA kit (# 515A, Orgentec Diagnostika®). Highly purified cardiolipin was coated on microwells with β2GPI. Specific antibodies in the patient sample bound to antigen. After a washing step, incubation with conjugate coupled to an enzyme was necessary. Unbound conjugates are washed out and a chromogenic substrate was added. The bound enzyme conjugate hydrolysed the substrate forming a blue coloured product. Finally, the product was detected with a microtiter plate reader (reader IRE 96, SFRI). According to the manufacturer's instructions, the cutoff for positivity was 10 U/ml for IgA and IgG and 7 U/ml for IgM.

### 2.3 Anti‐beta‐2 glycoprotein I antibodies assays

aβ2GPI‐IgG and IgM were evaluated by an ELISA kit (#521, Orgentec Diagnostika®) using a purified human β2GPI. aβ2GPI‐IgA were assessed by ELISA kit (#521A, Orgentec Diagnostika®). Following the manufacturer's instructions, the cutoff for positivity was 8 U/ml.

### 2.4 Detection of rheumatoid factors and anti‐cyclic citrullinated peptides antibodies

According to the manufacturer's instructions, RF IgG, IgM and IgA were assessed using a three commercially ELISA kits (#522G, #522 M and #522A respectively, Orgentec Diagnostika®).

CCP‐Ab were measured by an available second‐generation ELISA (Euroimmun®). Cutoff points were calculated by creating a receiver operating characteristic (ROC) curves. The ROC curves were created by plotting sensitivity as a function of 1‐specificity as described previously.^20^, ^21^

### 2.5 Detection of antinuclear antibodies

ANA were detected by indirect immunofluorescence on HEp‐2 cells (Euroimmun®). A titer ≥1:80 was considered as a positive result for the detection of ANA.

### 2.6 Statistical analysis

Statistical calculations were done by Statistical Package for Social Sciences version 22.0 (SPSS). Continuous variables were expressed as mean accompanied by standard deviation (SD) and discrete variables were expressed as percentages and numbers. Chi‐square test was used for comparisons of frequencies and Fischer's exact test was resorted when necessary. A *p* value bellow 0.05 was considered statistically significant.

## 3 RESULTS

Patient population was consisted of 250 females and 63 males (mean age: 49 ± 14 years; range: 17–87 years). For control group (212 females and 54 males), the mean age was 35 ± 11 years (Table 1). Arthritis was observed in 202 patients and arthralgia in 111.

**TABLE 1:** Characteristics of patients and the control group

	Patients (*n* = 313)	Control group (*n* = 266)	*p*
Sex‐ratio (F/M)	4 (250/63)	4 (212/54)	NS
Mean age (years)	49 ± 14	35 ± 11	<**10** ^**−6**^
Age range (years)	17–87	20–65	‐

*Note:* Bold values are statistically significant.

Abbreviations: F, female; M, male, NS, not significant.

The frequency of aPL (aCL and/or aβ2GPI) was significantly higher in patients than in HBD (24.9% vs. 10.9%; *p* < 10^−3^). Frequencies of aCL (IgG, IgM or IgA) were not different between patients and the control group. aβ2GPI were observed in 23.6% of patients and 9.4% of HBD and the difference between the two groups was statistically significant (*p* < 10^−3^). Compared to healthy population, patients had a significantly higher frequency of aβ2GPI‐IgA (20.4% vs. 7.5%, *p* < 10^−3^). aβ2GPI was most commonly observed than aCL in patients (23.6% vs. 6.4%, *p* < 10^−6^). Among the three isotypes of aβ2GPI tested, aβ2GPI‐IgA had the highest frequency (20.4%), followed by aβ2GPI‐IgG (5.4%) and aβ2GPI‐IgM (2.9%). Out of 64 patients with aβ2GPI‐IgA, 48 (75%) had only aβ2GPI‐IgA (isolated IgA; Table 2).

**TABLE 2:** Frequencies of aCL and aβ2GPI in patients and in the control group

Autoantibodies	Patients (*n* = 313) % (*n*)	Control group (*n* = 266) % (*n*)	*p*
aPL (aCL or aβ2GPI)	24.9 (78)	10.9 (29)	<**10** ^**−3**^
aCL (IgG, IgA or IgM)	6.4 (20)^a^	4.5 (12)	NS
aCL‐IgG	0.6 (2)	1.1 (3)	NS
aCL‐IgA	1.6 (5)	0.7 (2)	NS
aCL‐IgM	4.1 (13)	3.8 (10)	NS
aβ2GPI (IgG, IgA or IgM)	23.6 (74)^a^	9.4 (25)	<**10** ^**−3**^
aβ2GPI‐IgG	5.4 (17)^b^	2.6 (7)	NS
aβ2GPI‐IgA	20.4 (64)^b^ ^,^ ^c^	7.5 (20)	<**10** ^**−3**^
aβ2GPI‐IgM	2.9 (9)^c^	2.6 (7)	NS
Isolated aβ2GPI‐IgA	15.3 (48)	4.5 (12)	<**10** ^**−3**^

Bold value is statistically significant.

Abbreviations: aCL, anti‐cardiolipin antibodies; aPL, antiphospholipid antibodies; aβ2GPI, anti‐beta‐2 glycoprotein I antibodies; NS, not significant.

^a^ Comparison between aCL and aβ2GPI (*p* < **10** ^**−6**^).

^b^ Comparison between aβ2GPI‐IgG and aβ2GPI‐IgA (*p* < **10** ^**−6**^).

^c^ Comparison between aβ2GPI‐IgM and aβ2GPI‐IgA (*p* < **10** ^**−6**^).

**FIGURE 1:**
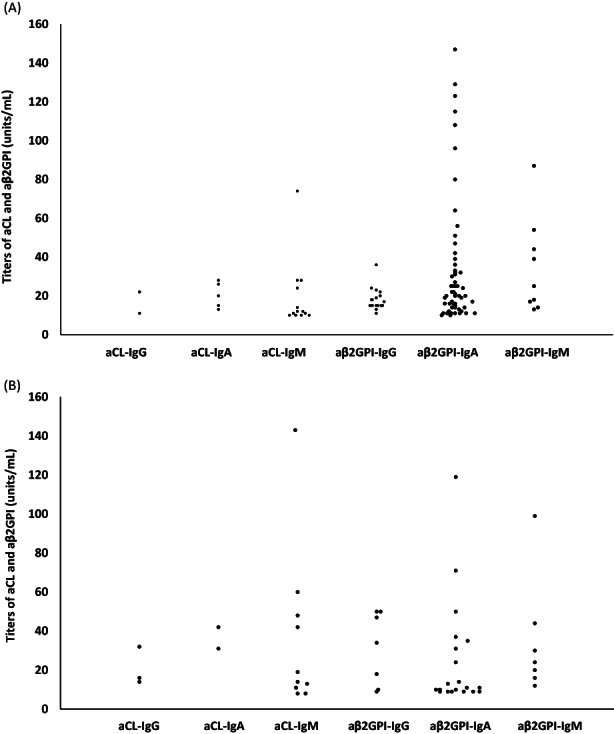
Titers of anticardiolipin antibodies and anti‐beta 2‐glycoprotein I antibodies in positive aPL patients (A) and control group (B). aCL, anticardiolipin antibodies; aβ2GPI, anti‐ beta 2‐glycoprotein I antibodies

Distribution of titers of aCL and aβ2GPI in positive aPL patients and control group was presented in Figure 1. Thirteen patients had moderate to high titers (≥40 units) of aβ2GPI‐IgA versus only three HBD (*p* = 0.02; Table 3).

**TABLE 3:** Frequencies of aPL with moderate to high titer (≥40 units) in patients and in the control group

Autoantibodies	Patients (*n* = 313) % (*n*)	Control group (*n* = 266) % (*n*)	*p*
aCL‐IgG	(0)	(0)	NS
aCL‐IgA	(0)	0.4 (1)	NS
aCL‐IgM	0.3 (1)	1.5 (4)	NS
aβ2GPI‐IgG	(0)	1.1 (3)	NS
aβ2GPI‐IgA	4.1 (13)	1.1 (3)	**0.02**
aβ2GPI‐IgM	0.9 (3)	0.7 (2)	NS

*Note:* Bold values are statistically significant.

Abbreviations: aβ2GPI, anti‐beta‐2 glycoprotein I antibodies; aPL, antiphospholipid antibodies; aCL, anti‐cardiolipin antibodies; NS, not significant.

In females, aPL were significantly more frequent in patients than in blood donors (24.4% vs. 9.9%, *p* < 10^−3^). aβ2GPI and aβ2GPI‐IgA positivity was more frequently observed in the female patients than in the healthy females (23.6% vs. 8%, *p* < 10^−3^; 20.8% vs. 6.6%, *p* < 10^−3^ respectively). In patients, the frequency of aPL was 24.4% in females and 27% in males, there was not statistically difference between the two frequencies (*p* = 0.67; Table 4).

**TABLE 4:** Frequencies of aPL according to sex

Autoantibodies	Females			Males		
	Patients (*n* = 250)	Control group (*n* = 212)	*p*	Patients (*n* = 63)	Control group (*n* = 54)	*p*
aPL % (*n*)	24.4 (61)	9.9 (21)	<**10** ^**−3**^	27 (17)	14.8 (8)	NS
aCL % (*n*)	5.2 (13)	4.2 (9)	NS	11.1 (7)	5.5 (3)	NS
aβ2GPI % (*n*)	23.6 (59)	8 (17)	<**10** ^**−3**^	23.8 (15)	14.8 (8)	NS
aβ2GPI‐IgA % (*n*)	20.8 (52)	6.6 (14)	<**10** ^**−3**^	19 (12)	11.1 (6)	NS

*Note:* Bold values are statistically significant.

Abbreviations: aβ2GPI, anti‐beta‐2 glycoprotein I antibodies; aCL, anti‐cardiolipin antibodies; aPL, antiphospholipid antibodies; NS, not significant.

## 4 DISCUSSION

Our study revealed a frequency of 24.9% of aPL. This frequency reflects those of aCL and aβ2GPI. Classification criteria for APS include not only aCL and aβ2GPI but also LA. LA has been described to be an independent risk factor for thrombosis.^22^ We did not perform LA and none of our patients had thrombosis at the time of sampling. Otherwise, the objective of this study was not to establish an association between thrombosis and aPL. By the way, in a study conducted by Azarsiz et al. including 100 children with different rheumatologic diseases, aβ2GPI IgG, IgA and IgM positivity did not show any relation to the presence of thrombosis.^23^

In the present study, positivity of aβ2GPI was 23.6% in patients with articular manifestations without evident cause. This frequency was high compared to that detected in the control group (9.4%). IgA was the most frequent isotype of aβ2GPI (20.4%) in the patient population. Interestingly, the frequency of isolated aβ2GPI‐IgA was 15.3%. Indeed, importance of IgA isotype directed against β2GPI has been increased over the last years. Moreover, many studies have identified these antibodies in diseases other than APS.^9^, ^10^, ^11^, ^12^, ^13^, ^14^, ^15^, ^16^, ^17^, ^18^ It is worth mentioning that IgA isotype of aβ2GPI was predominant in Africo‐Americans patients.^24^ Notably, our patients are from African origin and we have already demonstrated in previous studies that IgA was the main isotype of aβ2GPI in AID.^13^, ^15^, ^16^, ^25^

Several hypotheses are established to explain the synthesis of aβ2GPI‐IgA in patients with arthritis or arthralgia.

First, β2GPI is an anionic phospholipid‐binding glycoprotein that circulates in blood in an inactive closed‐circle conformation and most of the epitope recognized by aβ2GPI are cryptic.^26^ During chronic inflammatory arthritis, β2GPI present in closed conformation will be converted to an open hockey stick‐like conformation^27^ and cryptic epitopes are exposed and can be targeted by the aβ2GPI‐IgA. Furthermore, in our previous work carried out on 90 RA patients, we demonstrated that aβ2GPI‐IgA are frequent and could be implicated in the pathogenesis of arthritis in RA.^13^ It has even been suggested that aβ2GPI‐IgA predict coronary plaque progression in RA.^28^

Second, infections have been reported to trigger the production of aβ2GPI. Indeed, molecular mimicry between structure of the infectious agents and β2GPI molecule induce an autoimmune response and synthesis of aβ2GPI by a break‐down of the tolerance.^29^ Besides, we demonstrated, in our recent study, an elevated frequency of aβ2GPI‐IgA in patients with hepatitis C.^17^ Garcia‐Arellano et al.^30^ found that the most frequent aPL was aβ2GPI‐IgA on COVID‐19 patients with a frequency of 73.9%.

Third, joint pain and inflammation have been linked to microbiome intestinal composition. In fact, a report including 92 observational studies covering 14 rheumatic diseases suggested that dysbiosis in gut microbiota is associated with these diseases and could involve dysregulated immune responses, failure in immune tolerance and development of autoimmunity.^31^ Otherwise, it was shown greater abundance of *Streptococcus* species in patients with knee pain and this change in the composition of gastrointestinal microbiome was explained by the local inflammatory process in joints.^32^ Maeda et al.^33^ demonstrated that intestinal microbiota dysbiosis boosts the sensitivity to arthritis. An altered composition of microbiota in the intestine predominated by *Prevotella* spp initiates the activation of autoreactive T cells. These cells interact with autoantigens, in particular, arthritis‐related antigens inducing joint inflammation. Intriguingly, Ruff et al.^34^ hypothesized that gut microbiota can trigger the generation of aβ2GPI by several mechanisms in genetically predisposed individuals. Cross‐reactive gut commensal antigens may promote break in tolerance and induce β2GPI‐directed response. Indeed, lipopolysaccharides (LPS) and phospholipides derived from gut commensals lead to a conformational change which reveals cryptic epitopes of β2GPI in domains I and V.^34^

It was reported that high titer of aPL is an important risk factor for thrombosis development, whereas low titers of aPL may be transient and observed frequently in infections, and medications or cancer.^8^ In the present study, patients were more likely to have moderate to elevated titers of aβ2GPI‐IgA than HBD but our patients had no thrombosis, so aβ2GPI‐IgA could have a pathogenic role in manifestations other than those described in APS. Consequently, we wondered if IgA of aβ2GPI are implicated in the development of arthritis or arthralgia.

In one hand, β2GPI protein belongs to the complement control superfamily and is implicated in the regulation of the clotting cascade. Recently, new insights have been suggested for the role of β2GPI as a scavenger protein implicated in the apoptotic cells and microorganisms clearance from the circulation and also in the removal of inflammatory and prothrombotic cell debris by macrophages.^35^ When aβ2GPI‐IgA bind to their specific antigen, this last will be blocked resulting in an inhibiting effect of all its functions. A consequent inflammatory state can set in. Furthermore, aβ2GPI enhance complement activation due to the fact that β2GPI molecule can be a mimetic of CR1. So, aβ2GPI can shadow complement inhibitor and increase complement activation.^36^ Also, aβ2GPI neutralize the C4bp, enabling an increase in complement activation and therefore inflammation.^36^ As β2GPI is an ubiquitous protein that exists in joints,^27^ aβ2GPI can migrate to joints to link to their target antigen causing inflammation and therefore arthritis.

On the other hand, among consequence of aβ2GPI‐IgA production, we can cite cytokine and inflammatory mediators release.^35^ The resulting inflammatory state can serve as a “second hit” and antibodies can convey their pathogenic potential. In fact, the “second hit theory” proposed by Meroni^37^ was developed to explain how some patients with aPL developed symptoms, while others remain asymptomatic. According to this theory, the “second hit” could be an inflammatory stimulus and is needed for the development of clinical manifestations.^35^

Some limitations must be highlighted. First, LA had not been done. Second, the cutoffs of aPL positivity used were those provided by the manufacturer, and this could explain the high frequency of aPL in the control group (10.9%). In fact, the manufacturer's cutoffs were determined on a healthy population of different geographical origin. Third, we did not have access to a second sample to confirm aPL positivity after baseline screening. Lastly, the samples of the control group were not tested for serological markers of RA but to our knowledge, none of the HBD suffered from arthralgia or arthritis.

As far as we know, this is the first study demonstrating an elevated frequency of aβ2GPI‐IgA in patients with unexplained arthralgia or arthritis. The possible pathogenic mechanism of aβ2GPI remained to be demonstrated and a prospective study is necessary to known if aβ2GPI‐IgA will persist over time.

## CONFLICT OF INTEREST

None of the authors have conflicts of interest to declare.

## Data Availability

The data supporting the finding of the study are available from the corresponding author up on reasonable request.

## References

[1] Asherson RA , Cervera R , Merrill JT , Erkan D . Antiphospholipid antibodies and the antiphospholipid syndrome: clinical significance and treatment. Semin Thromb Hemost. 2008;34(3):256‐266. doi:10.1055/s-0028-1082269 18720305

[2] Garcia D , Erkan D . Diagnosis and management of the antiphospholipid syndrome. N Engl J Med. 2018;378(21):2010‐2021. doi:10.1056/NEJMra1705454 29791828

[3] Miyakis S , Lockshin MD , Atsumi T , et al. International consensus statement on an update of the classification criteria for definite antiphospholipid syndrome (APS). J Thromb Haemost. 2006;4(2):295‐306. doi:10.1111/j.1538-7836.2006.01753.x 16420554

[4] Meroni PL , Borghi MO . Antiphospholipid antibody assays in 2021: looking for a predictive value in addition to a diagnostic one. Front Immunol. 2021;12:726820. doi:10.3389/fimmu.2021.726820 34621272 PMC8490700

[5] Reshetnyak T , Cheldieva F , Cherkasova M , Lila A , Nasonov E . IgA antiphospholipid antibodies in antiphospholipid syndrome and systemic lupus erythematosus. Int J Mol Sci. 2022;23(16):9432‐9444. doi:10.3390/ijms23169432 36012697 PMC9409442

[6] Murthy V , Willis R , Romay‐Penabad Z , et al. Value of isolated IgA anti‐β2 ‐glycoprotein I positivity in the diagnosis of the antiphospholipid syndrome. Arthritis Rheum. 2013;65(12):3186‐3193. doi:10.1002/art.38131 23983008 PMC4048705

[7] Yang B , Zhao JL , Huang ZC , et al. Value of IgA antiphospholipid antibodies in diagnosis of the antiphospholipid syndrome. Zhonghua Yi Xue Za Zhi. 2021;101(41):3404‐3410. doi:10.3760/cma.j.cn112137-20210424-00976 34758544

[8] Su Z , Huang Z , Zhao J , et al. Detection of IgA antiphospholipid antibodies does not improve thrombotic antiphospholipid syndrome classification: a two‐center study. Clin Appl Thromb Hemost. 2022;28:1‐9. doi:10.1177/10760296221081129 PMC8988664 35379020

[9] Staub HL , Franck M , Ranzolin A , Norman GL , Iverson GM , von Mühlen CA . IgA antibodies to beta2‐glycoprotein I and atherosclerosis. Autoimmun Rev. 2006;6(2):104‐106. doi:10.1016/j.autrev.2006.06.014 17138253

[10] Morales JM , Martinez‐Flores JA , Serrano M , et al. Association of early kidney allograft failure with preformed IgA antibodies to β2‐glycoprotein I. J Am Soc Nephrol. 2015;26(3):735‐745. doi:10.1681/ASN.2014030228 25071084 PMC4341482

[11] Delgado JF , Serrano M , Morán L , et al. Early mortality after heart transplantation related to IgA anti‐β2‐glycoprotein I antibodies. J Heart Lung Transplant. 2017;36(11):1258‐1265. doi:10.1016/j.healun.2017.05.016 28579112

[12] Serrano A , García F , Serrano M , et al. IgA antibodies against β2 glycoprotein I in hemodialysis patients are an independent risk factor for mortality. Kidney Int. 2012;81(12):1239‐1244. doi:10.1038/ki.2011.477 22358146

[13] Melayah S , Changuel M , Mankaï A , Ghedira I . IgA is the predominant isotype of anti‐β2 glycoprotein I antibodies in rheumatoid arthritis. J Clin Lab Anal. 2020;34(6):e23217. doi:10.1002/jcla.23217 31967351 PMC7307372

[14] Gabeta S , Norman GL , Gatselis N , et al. IgA anti‐b2GPI antibodies in patients with autoimmune liver diseases. J Clin Immunol. 2008;28(5):501‐511. doi:10.1007/s10875-008-9211-6 18551357

[15] Mankaï A , Achour A , Thabet Y , Manoubia W , Sakly W , Ghedira I . Anti‐cardiolipin and anti‐beta 2‐glycoprotein I antibodies in celiac disease. Pathol Biol (Paris). 2012;60(5):291‐295. doi:10.1016/j.patbio.2011.07.003 21839587

[16] Mankaï A , Manoubi W , Ghozzi M , Melayah S , Sakly W , Ghedira I . High frequency of antiphospholipid antibodies in primary biliary cirrhosis. J Clin Lab Anal. 2015;29(1):32‐36. doi:10.1002/jcla.21723 24687920 PMC6807226

[17] Melayah S , Kallala O , Ben Ahmed M , et al. IgA anti‐beta‐2 glycoprotein I antibodies in chronic hepatitis C. Arab J Gastroenterol. 2022;23(1):26‐31. doi:10.1016/j.ajg.2021.12.003 35123900

[18] Hasan Ali O , Bomze D , Risch L , et al. Severe coronavirus disease 2019 (COVID‐19) is associated with elevated serum immunoglobulin (Ig) a and antiphospholipid IgA antibodies. Clin Infect Dis. 2021;73(9):e2869‐e2874. doi:10.1093/cid/ciaa1496 32997739 PMC7543315

[19] Krustev E , Rioux D , McDougall JJ . Mechanisms and mediators that drive arthritis pain. Curr Osteoporos Rep. 2015;13(4):216‐224. doi:10.1007/s11914-015-0275-y 26025232

[20] Sghiri R , Bouagina E , Zaglaoui H , et al. Diagnostic performances of anti‐cyclic citrullinated peptide antibodies in rheumatoid arthritis. Rheumatol Int. 2007;27(12):1125‐1130. doi:10.1007/s00296-007-0351-4 17447069

[21] Sghiri R , Bouajina E , Bargaoui D , et al. Value of anti‐mutated citrullinated vimentin antibodies in diagnosing rheumatoid arthritis. Rheumatol Int. 2008;29(1):59‐62. doi:10.1007/s00296-008-0614-8 18496693

[22] Ruffatti A , Del Ross T , Ciprian M , et al. Risk factors for a first thrombotic event in antiphospholipid antibody carriers: a prospective multicentre follow‐up study. Ann Rheum Dis. 2011;70(6):1083‐1086. doi:10.1136/ard.2010.142042 21285115

[23] Azarsiz E , Eman G , Akarcan SE , et al. Antı‐β2 glycoprotein I antibodies in children with rheumatologic disorders. Indian J Clin Biochem. 2019;34(1):95‐100. doi:10.1007/s12291-017-0711-0 30728679 PMC6346611

[24] Diri E , Cucurull E , Gharavi AE , et al. Antiphospholipid (Hughes') syndrome in African‐Americans: IgA aCL and abeta2 glycoprotein‐I is the most frequent isotype. Lupus. 1999;8(4):263‐268. doi:10.1191/096120399678847812 10413203

[25] Mankaï A , Sakly W , Thabet Y , Achour A , Manoubi W , Ghedira I . Anti‐Saccharomyces cerevisiae antibodies in patients with systemic lupus erythematosus. Rheumatol Int. 2013;33(3):665‐669. doi:10.1007/s00296-012-2431-3 22527140

[26] de Groot PG , Meijers JC . β(2) ‐glycoprotein I: evolution, structure and function. J Thromb Haemost. 2011;9(7):1275‐1284. doi:10.1111/j.1538-7836.2011.04327.x 21535391

[27] Pawlak Z , Mrela A , Kaczmarek M , Cieszko M , Urbaniak W . Natural joints: boundary lubrication and antiphospholipid syndrome (APS). Biosystems. 2019;177:44‐47. doi:10.1016/j.biosystems.2018.10.018 30389556

[28] Karpouzas GA , Ormseth SR , Hernandez E , Bui VL , Budoff MJ . Beta‐2‐glycoprotein‐I IgA antibodies predict coronary plaque progression in rheumatoid arthritis. Semin Arthritis Rheum. 2021;51(1):20‐27. doi:10.1016/j.semarthrit.2020.10.003 33360226

[29] Blank M , Shoenfeld Y . Beta‐2‐glycoprotein‐I, infections, antiphospholipid syndrome and therapeuticconsiderations. Clin Immunol. 2004;112(2):190‐199. doi:10.1016/j.clim.2004.02.018 15240163

[30] Garcia‐Arellano G , Camacho‐Ortiz A , Moreno‐Arquieta IA , et al. Anticardiolipin and anti‐beta‐2 glycoprotein I antibodies in patients with moderate or severe COVID‐19. Am J Med Sci. 2022:S0002‐9629(22)00453‐0. doi:10.1016/j.amjms.2022.10.012 PMC9632263 36335994

[31] Wang Y , Wei J , Zhang W , et al. Gut dysbiosis in rheumatic diseases: a systematic review and meta‐analysis of 92 observational studies. EBioMedicine. 2022;80:104055. doi:10.1016/j.ebiom.2022.104055 35594658 PMC9120231

[32] Boer CG , Radjabzadeh D , Medina‐Gomez C , et al. Intestinal microbiome composition and its relation to joint pain and inflammation. Nat Commun. 2019;10(1):4881‐4890. doi:10.1038/s41467-019-12873-4 31653850 PMC6814863

[33] Maeda Y , Kurakawa T , Umemoto E , et al. Dysbiosis contributes to arthritis development via activation of autoreactive T cells in the intestine. Arthritis Rheumatol. 2016;68(11):2646‐2661. doi:10.1002/art.39783 27333153

[34] Ruff WE , Vieira SM , Kriegel MA . The role of the gut microbiota in the pathogenesis of antiphospholipid syndrome. Curr Rheumatol Rep. 2015;17(1):472‐489. doi:10.1007/s11926-014-0472-1 25475595 PMC4394866

[35] Cabrera‐Marante O , Rodríguez de Frías E , Serrano M , et al. The weight of IgA anti‐β2glycoprotein I in the antiphospholipid syndrome pathogenesis: closing the gap of seronegative antiphospholipid syndrome. Int J Mol Sci. 2020;21(23):8972‐8995. doi:10.3390/ijms21238972 33255963 PMC7730063

[36] Blank M , Asherson RA , Cervera R , Shoenfeld Y . Antiphospholipid syndrome infectious origin. J Clin Immunol. 2004;24(1):12‐23. doi:10.1023/B:JOCI.0000018058.28764.ce 14997029

[37] Meroni PL , Chighizola CB , Rovelli F , Gerosa M . Antiphospholipid syndrome in 2014: more clinical manifestations, novel pathogenic players and emerging biomarkers. Arthritis Res Ther. 2014;16(2):209‐223. doi:10.1186/ar4549 25166960 PMC4060447

